# It is time to change the way we manage mild asthma: an update in GINA 2019

**DOI:** 10.1186/s12931-019-1159-y

**Published:** 2019-08-14

**Authors:** Jaya Muneswarao, Mohamed Azmi Hassali, Baharudin Ibrahim, Bandana Saini, Irfhan Ali Hyder Ali, Ashutosh Kumar Verma

**Affiliations:** 1 School of Pharmaceutical Sciences, Universiti Sains Malaysia, Minden, Penang Malaysia; 2 School of Pharmacy, The University of Sydney, Sydney, New South Wales Australia; 3 Respiratory Department, Hospital Pulau Pinang, Ministry of Health Malaysia, George Town, Penang Malaysia; 4 Department of Pharmacy, IEC Group of Institution, Greater Noida, India

**Keywords:** Global Initiative for Asthma (GINA), Mild asthma, Symptom-driven, Short-acting β_2_ agonists (SABA) overuse

## Abstract

Asthma is a heterogeneous lung disease, usually characterised by chronic airway inflammation. Although evidence-based treatments are available in most countries, asthma control remains suboptimal, and asthma-related deaths continue to be an ongoing concern. Generally, it is believed that between 50 to 75% of patients with asthma can be considered as having mild asthma.

Previous versions of Global Initiative for Asthma (GINA) suggested that mild asthma in adults can be well managed with either reliever medications, for example, short-acting beta_2_ agonists (SABA) alone or with the additional use of controllers such as regular low-dose inhaled corticosteroids (ICS). Given the low frequency or non-bothersome nature of symptoms in mild asthma, patients’ adherence towards their controller medications, especially to ICS is usually not satisfactory. Such patients often rely on SABA alone to relieve symptoms, which may contribute to SABA over-reliance. Overuse of relievers such as SABAs has been associated with poor asthma outcomes, such as exacerbations and even deaths. The new GINA 2019 asthma treatment recommendations represent significant shifts in asthma management at Steps 1 and 2 of the 5 treatment steps. The report acknowledges an emerging body of evidence suggesting the non-safety of SABAs overuse in the absence of concomitant controller medications, therefore does not support SABA-only therapy in mild asthma and has included new off-label recommendations such as symptom-driven (as-needed) low dose ICS-formoterol and “low dose ICS taken whenever SABA is taken”.

The GINA 2019 report highlights significant updates in mild asthma management and these recommendations represent a clear deviation from decades of clinical practice mandating the use of symptom-driven SABA treatment alone in those with mild asthma. While the new inclusions of strategies such as symptom-driven (as-needed) ICS-formoterol and “ICS taken whenever SABA is taken” are based on several key trials, data in this context are still only emergent data, with clear superiority of as needed ICS-formoterol combinations over maintenance ICS regimens yet to be established for valid endpoints. Nevertheless, current and emerging data position the clinical asthma realm at a watershed moment with imminent changes for the way we manage mild asthma likely in going forward.

## Background

Asthma is a heterogeneous lung disease, usually characterised by chronic airway inflammation [1]. Asthma poses a significant level of morbidity and mortality globally [1, 2]. Although evidence-based treatments are available in most countries, asthma control remains suboptimal, and asthma-related deaths continue to be an ongoing concern [3, 4]. Generally, it is believed that between 50 to 75% of patients with asthma can be considered as having mild asthma [5, 6]. Although the symptoms may not be very troublesome or frequent, airway inflammation is usually present in those with mild asthma and patients may be at risk of acute asthma exacerbations and death [6–8]. An expert review estimated that the frequency of severe exacerbations in mild asthma ranged between 0.12 to 0.77 per patient-year [6]. Further, the review highlighted that between 30 and 40% of exacerbations requiring emergency care appear to be in patients with mild asthma [6]. In general, asthma exacerbations impose a significant disease burden, including hospitalisation, a greater progressive decline in lung function, impairment in quality of life (QoL) and death [9–13]. Moreover, asthma patients with exacerbations requiring an emergency department visit or hospitalisation are at increased risk for future exacerbations, independent of demographic, clinical factors, asthma severity and asthma control [14]. Asthma exacerbations also have been shown to cause a significant financial burden on health systems [15]. For example, in a US study which retrospectively analysed administrative claims data, asthma patients who experienced exacerbations had nearly twice the health care and asthma specific costs compared with patients without exacerbations [16]. Preventing the risk of future exacerbations is, therefore, an important target, particularly in patients with mild asthma, where there may be complacence both by the patient or their health professionals.

## Discussion

Despite concerns, previous versions of the treatment recommendations from the Global Initiative for Asthma (GINA) suggested that mild asthma in adults can be well managed with either reliever medications, for example, short-acting beta_2_ agonists (SABA) alone (used ‘as-needed’) or with the additional use of controllers such as regular low-dose inhaled corticosteroids (ICS). Given the low frequency or non-bothersome nature of symptoms in mild asthma, patients’ adherence towards their controller medications, especially to ICS is usually not satisfactory [17–19]. Such patients often rely on SABAs alone to relieve symptoms, which may contribute to SABA overuse [17]. This may particularly occur if SABAs are available in pharmacies as non-prescription medicines as is the case, for example, in Australia [20], but is a prevalent issue even in countries where SABAs are available only on prescription [21, 22]. In people with mild asthma, the perception of quick-relief evident when SABAs are used may also contribute to over-reliance on SABAs and other similar relievers, compared to controllers where the actions of the medications are not immediately perceivable. Overuse of relievers such as SABAs has been associated with poor asthma outcomes, such as exacerbations and even deaths [8, 23, 24].

The new GINA 2019 asthma treatment recommendations represent significant shifts in asthma management at Steps 1 and 2 of the 5 treatment steps [25]. These changes can be thought of as revolutionising mild asthma patient management and new recommendations acknowledge an emerging body of evidence suggesting the non-safety of SABAs overuse in the absence of concomitant controller medications. The present article is focused on recommendations in the adolescent and adult population with mild asthma. The differences between GINA 2018 and 2019 treatment recommendations are summarised in Table 1. The summary of the clinical trials supporting these recommendations are listed in Tables 2 and 3.

**Table 1:** Differences in recommended Step 1 and 2 controller options between GINA 2018 and 2019 [1, 25]

	Controller options	GINA 2018	GINA 2019
Step 1 *(Patients with symptoms <twice a month and no exacerbation risk factors)*	Preferred	• SABA as-needed and no controller.	• **As-needed low dose ICS-formoterol (off- label).**
	Other options	• Daily low dose ICS.	• **Low dose ICS taken whenever SABA is taken (off-label).** This may involve combination (ICS-SABA) in a single or separate (ICS inhaler + SABA inhaler) inhaler/s.
Step 2	Preferred	• Daily low dose ICS.	• Daily low dose ICS. • **As-needed low dose ICS-formoterol (off-label)**
	Other options	• Daily LTRA. • Daily low dose ICS-LABA.	• **Low dose ICS taken whenever SABA is taken (off-label)** • Daily LTRA. • Daily low dose ICS-LABA (better improvement in symptoms and FEV_1_ than when ICS is used alone but more costly, and exacerbation rate is similar to the above option).

*SABA = Short-acting beta*_*2*_ *agonist, LABA = Long-acting beta*_*2*_ *agonist, ICS=Inhaled corticosteroids, LTRA = Leukotriene receptor antagonist, FEV*_*1*_ *= Forced expiratory volume in one second*

*Note:* (1) *The new recommendations in GINA 2019 are highlighted in bold* (2) *The reliever option in GINA2019 is as-needed low dose ICS-formoterol or as-needed SABA*

**Table 2:** The summary of clinical trials supporting the recommendation for symptom-driven (as-needed) ICS-formoterol strategy

Name of the trial	Symbicort Given as Needed in Mild Asthma 1 (SYGMA 1) [5]	Symbicort Given as Needed in Mild Asthma 2 (SYGMA 2) [17]
Trial Design	Double-blind, multisite, parallel-group RCT (Phase 3 trial)	Double-blind, multisite, parallel-group RCT (Phase 3 trial)
Trial Duration	52 weeks	52 weeks
Patient population	1. Inclusion criteria: > 12 years or older, diagnosed with mild asthma at least 6 months previous to trial and deemed as needing Step 2 treatment 2. Average age of included patients: 39.6 ± 16.6 years.	1. Inclusion criteria: > 12 years or older, diagnosed with mild asthma at least 6 months previous to trial and deemed as needing Step 2 treatment 2. Average age of included patients: 41.0 ± 17.0 years
Total number of patients	3849	4215
Treatment arms	1. Twice-daily placebo + as-needed terbutaline (0.5 mg). 2. Twice-daily placebo + as-needed budesonide-formoterol (200/6 μg). 3. Twice-daily budesonide (200 μg) + as-needed terbutaline (0.5 mg).	1. Twice-daily placebo + as-needed budesonide-formoterol (200/6 μg). 2. Twice-daily budesonide (200 μg) + as-needed terbutaline (0.5 mg).
Primary outcome	Weeks with well-controlled asthma.	Annualised rate of severe exacerbations
Conclusion	As-needed inhaled budesonide-formoterol provided superior asthma-symptom control to as-needed terbutaline but was inferior to budesonide maintenance therapy. Exacerbation rates with the two budesonide-containing regimens were similar and lower than in the terbutaline only group. Budesonide-formoterol used as-needed, resulted in substantially lower glucocorticoid exposure.	As-needed use of inhaled budesonide-formoterol was non-inferior to budesonide maintenance therapy concerning the annualised rate of severe asthma exacerbations but was inferior in controlling symptoms. Budesonide-formoterol used as-needed, resulted in substantially lower glucocorticoid exposure.

*RCT* Randomised controlled trial

**Table 3:** The summary of clinical trials supporting the recommendation for symptom-driven (as-needed) ICS with SABA strategy

Name of the trial	BEST (Beclomethasone plus Salbutamol Treatment) [33]	BASALT (Best Adjustment Strategy for Asthma in the Long Term) [35]	TREXA (Treating Children to Prevent Exacerbations of Asthma) [34]
Trial Design	Double-blind, double-dummy, randomised parallel group trial	Multiple blind, parallel, 3-group, randomised, placebo-controlled trial	Double-blind, four-treatment, placebo-controlled, randomised, parallel group trial
Trial Duration	6 months	9 months	44 weeks
Patient population	18–65 years and diagnosed with mild persistent asthma	> 18 years and diagnosed with mild to moderate persistent asthma well controlled with low-dose inhaled corticosteroids	6–18 years and diagnosed with mild persistent asthma.
Total number of patients	466	342	843
Treatment arms	1. Twice-daily placebo + as-needed beclometasone (250 μg) with salbutamol (100 μg) in a single inhaler. 2. Twice-daily placebo + as-needed salbutamol (100 μg). 3. Twice-daily beclometasone (250 μg) + as-needed salbutamol (100 μg). 4. Twice-daily beclometasone (250 μg) and salbutamol (100 μg) in a single inhaler + as-needed salbutamol (100 μg).	1. Beclometasone dose adjusted using physician assessment-based approach (PABA). 2. Beclometasone dose adjusted using biomarker-based (FeNO) approach (BBA). 3. Beclometasone dose adjusted based on symptoms (need for salbutamol), i.e. a symptom-based approach (SBA)	1. Twice-daily beclomethasone (40 μg) + as-needed beclometasone (40 μg) with salbutamol (90 μg). 2. Twice-daily beclomethasone (40 μg) + as-needed placebo with salbutamol (90 μg). 3. Twice-daily placebo + as-needed beclometasone (40 μg) with salbutamol (90 μg). 4. Twice-daily placebo + as-needed placebo with salbutamol (90 μg)
Primary outcome	Morning peak expiratory flow rate	Time to first treatment failure	Time to first exacerbation requiring a prednisone dose
Conclusion	Symptom-driven use of ICS with SABA in a single inhaler is as effective as ICS maintenance therapy and is associated with a lower cumulative dose of the ICS	Neither the SBA nor the BBA strategy for ICS therapy was superior to the standard PABA strategy for the outcome of treatment failure. Mean monthly inhaled beclometasone dose was lowest in the SBA group.	Daily ICS was the most effective treatment to prevent exacerbations. As-needed ICS with SABA was more effective at reducing exacerbations compared with SABA alone and had the lowest daily ICS dose. Rescue treatment with SABA alone should be avoided.

*ICS* Inhaled corticosteroid, *SABA* Short-acting beta_2_ agonist

SABAs are highly effective bronchodilators with a quick onset of action, allowing immediate relief of symptoms associated with bronchoconstriction and are crucial in acute asthma management [26]. However, earlier studies have shown that higher usage of SABAs in the absence of effective anti-inflammatory treatment, was associated with increased risk of asthma exacerbations, hospital admissions and asthma-related deaths as well as amplified levels of airway inflammation [23, 24, 27–30]. Evidence suggests that these adverse associations of SABAs are not necessarily a result of the direct actions of the drugs, but because they may be used preferentially by patients instead of regular ICS or ICS combinations with long-acting β2 agonists (LABAs) and may mask worsening asthma symptoms [26]. Therefore, the new GINA 2019 strategy report does not support SABA-only therapy at Step 1 treatment level [25]. The preferred controller treatment at the Step 1 level stated in the 2019 document comprises as-needed low dose ICS-formoterol (off-label) where this combination serves as a ‘reliever’ as well.

The GINA treatment recommendations for controller treatment are based on evidence generated in several trials. The first of these was the three-way Symbicort Given As Needed in Mild Asthma (SYGMA) 1 trial (Table 2) which included patients deemed to be on GINA Step 2 treatment [5, 25]. The trial results indicated that as-needed budesonide-formoterol combinations provided a similar (non-inferior) effect on annual rate of exacerbation reduction, and eventuated in a lower exposure to ICSs compared to a maintenance ICS regimen – although budesonide-formoterol used as needed was inferior in terms of conferring ongoing asthma control (proportion of weeks with good asthma control) [5]. Both as-needed ICS-formoterol and maintenance ICS groups, were, as expected, better performing than terbutaline only group [5]. Another study, the SYGMA 2 trial randomised asthma patients deemed to be requiring GINA Step 2 to treatment with either twice-daily placebo plus as-needed budesonide-formoterol or twice-daily budesonide plus as-needed SABA for 52 weeks [17]. The results of this trial also indicated non-inferiority of the as-needed budesonide-formoterol combination compared to the maintenance ICS plus as-needed SABA regimen in reducing the annual severe exacerbation rate in patients with mild asthma [17]. The findings indicated significantly more positive results for the maintenance versus as-needed regimen for asthma symptom control, asthma-related QoL and lung function, however, these differences in outcomes were not deemed as clinically important [5, 17, 25]. Recent updates from the Novel START (Novel Symbicort Turbuhaler Asthma Reliever Therapy) trial support the findings from the SYGMA 1 and 2 trials. The Novel START randomised mild asthma patients to either 1. as-needed salbutamol; 2. twice-daily budesonide plus as-needed salbutamol; or 3. as-needed budesonide-formoterol in an open-label, parallel-group, randomised controlled trial which included 668 patients with mild asthma [31]. Adult patients from 18 to 75 years of age were recruited in this study and the trial duration was 52 weeks. The primary outcome was the annualised rate of asthma exacerbations. The exacerbation rate among patients treated with as-needed budesonide-formoterol was significantly lower compared with patients treated with as-needed SABA and did not differ significantly from patients who received twice-daily maintenance budesonide. However, maintenance treatment with budesonide was superior to as-needed budesonide-formoterol in terms of asthma symptom control, measured by Asthma Control Questionnaire-5 (ACQ-5) [31]. The results of the PRACTICAL (PeRsonalised Asthma Combination Therapy with an Inhaled Corticosteroid And fast-onset Long acting beta agonist) trial, which is investigating the effectiveness of as-needed budesonide-formoterol in real-world settings (several key differences in trial design compared with Novel START) are also awaited with great interest [32].

The SYGMA 1 and 2 trials compared as-needed versus maintenance regimens for the budesonide-formoterol combination, however, there are other ICS-formoterol combination/s (beclometasone-formoterol) available on the market which may potentially be used in a similar fashion [25]. Although the results in terms of ongoing asthma symptom control were slightly better in the SYGMA trials for the maintenance regimen of daily low dose budesonide compared with the as-needed regimen of budesonide-formoterol, adherence to regular ICS is considered an issue in patients with very mild asthma requiring only Step 1 treatment. Non-adherent patients may thus be exposed to SABAs alone if they are prescribed regular ICSs. Further, the use of as-needed ICS-formoterol also has the advantage of lower exposure to ICSs [25], for example, a similar rate of lowering in the annualised rate of exacerbations was achieved in the SYGMA 2 trial with one fourth the level of ICS exposure in the as-needed regimen arm compared with the maintenance regimen arm [17]. Based on this reasoning, daily ICS regimens are no longer listed in Step 1 under the ‘other controller option’ in GINA 2019 recommendations. Instead, the recommendations suggest an ICS inhaler used whenever an ‘as- needed’ SABA inhaler is used (Table 1) [25]. The evidence for this regimen (using an ICS inhaler whenever an as-needed SABA is used) is again indirect and extrapolated from trials such as the BEST (Beclomethasone plus Salbutamol Treatment) [33], TREXA (Treating Children to Prevent Exacerbations of Asthma) [34] and BASALT (Best Adjustment Strategy for Asthma in the Long Term) [35], which recruited patients deemed to be requiring treatment in Step 2 (Table 3). Concerning this recommendation, high importance was given to preventing severe exacerbations, and lower emphasis was given to small differences in symptom control and the inconvenience of carrying two inhalers [25].

For patients requiring Step 2 treatment, GINA 2019 has retained the previous recommendation for preferred controller treatment as daily low dose ICS with as-needed SABA [25]. This is based on cumulative evidence demonstrating that regular low dose ICS use substantially reduces asthma symptoms, increases lung function, improves QoL and reduces risks of severe exacerbations, hospitalisations or death [1, 36]. The benefits of low dose ICS are evident even in mild asthma [36]. Another preferred controller option in Step 2 in the 2019 GINA recommendations is the newly included, as-needed low dose ICS-formoterol (off-label) combination which reflects the clinical concern of non-adherence to regular low dose ICSs in people with milder forms of asthma (needing Step 1 and Step 2 treatment) and resultant exposure to SABA monotherapy with such non-adherence [25]. GINA 2019 also added the new recommendation “low dose ICS taken whenever SABA is taken” (off-label, combination/separate inhalers) in Step 2 management (under other controller options) [25].

It should be acknowledged that the newly recommended symptom-driven or as-needed (preferred option) treatment recommendation in GINA 2019 was only based on 2 budesonide-formoterol trials (designed to establish superiority against SABA monotherapy in SIGMA 1 and non-inferiority compared with regular ICS in both SYGMA trials) [5, 17], this was the best available evidence at the point of recommendation; since then findings from the Novel START trial [31] also support these recommendations. There are still a few issues that are still unclear, for example, the long term impact of these strategies on airway inflammation, hyper-responsiveness, remodelling and asthma mortality compared with regular ICS usage is as yet unknown. An editorial in the European Respiratory Journal highlighted several practical issues before the strategies to stop SABA monotherapy use could be implemented [26]. Drug availability and regulatory indications (e.g. ICS-formoterol licensed only for maintenance use) may serve as limitations in some countries. [26]. Another concern is that the role of ICS-formoterol or “ICS use whenever a SABA is used” in situations where SABA monotherapy is currently used, such as acute exacerbation of asthma is as yet unevaluated [26]. The publication of the Steroids in Eosinophil Negative Asthma (SIENA) trial further adds to the controversies [37]. Lazarus et al. conducted a 42-week, three-period, randomised, double-blind, placebo-controlled crossover trial which recruited patients who were at least 12 years of age and had mild persistent asthma. The patients were classified according to the sputum eosinophil (Eos) level (high if sputum Eos ≥2% or low if sputum Eos ≤2%) [37]. The patients received mometasone (via Twisthaler or pMDI), tiotropium (via Respimat), or placebo. The primary outcome was the response to mometasone as compared with placebo and to tiotropium as compared with placebo among patients with a low Eos level. A composite outcome was used which included treatment failure, asthma-control days, and FEV_1_ [37]. Among the patients with a low Eos level, the percentage of patients who had a better response to mometasone (57%) than to placebo was not significantly different from the percentage who had a better response to tiotropium (60%) than to placebo. In the high Eos group, ICS performed significantly better [37]. Although the SIENA trial had a small sample and a relatively short follow up, the results provide a signal towards the consideration of phenotype-based treatment rather than blanket recommendations, i.e. for low dose ICS for all patients with mild asthma [38].

## Conclusion

The new GINA 2019 report highlights significant updates in mild asthma management and these recommendations represent a clear deviation from decades of clinical practice mandating the use of symptom-driven SABA treatment alone in those with mild asthma. While the new inclusions of strategies such as symptom-driven (as-needed) ICS-formoterol and “ICS taken whenever SABA is taken” are based on several key trials, data in this context are still only emergent data, with clear superiority of as needed ICS-formoterol combinations over maintenance ICS regimens yet to be established for valid endpoints. There also remain several issues relating to implementations of these strategies globally and their long-term effects in mild asthma patients. Further discussions are needed on this matter. It also remains to be observed if other guidelines such as (such as the British Thoracic Society and National Heart, Lung, and Blood Institute) share the same vision with GINA in their updates/revisions. Nevertheless, current and emerging data position the clinical asthma realm at a watershed moment with imminent changes for the way we manage mild asthma likely in going forward.

## Data Availability

Data sharing does not apply to this article as no datasets were generated or analysed during the current study.

## Abbreviations

BASALT: Best Adjustment Strategy for Asthma in the Long Term
BEST: Beclomethasone plus Salbutamol Treatment
FEV_1_: Forced expiratory volume in one second
GINA: Global Initiative for Asthma
ICS: Inhaled corticosteroids
LABA: Long-acting beta_2_ agonists
Novel START: Novel Symbicort Turbuhaler Asthma Reliever Therapy
PRACTICAL: PeRsonalised Asthma Combination Therapy with an Inhaled Corticosteroid And fast-onset Long acting beta agonist
QoL: Quality of life
RCT: Randomised controlled trial
SABA: Short-acting beta_2_ agonists
SIENA: Steroids in Eosinophil Negative Asthma
SYGMA: Symbicort Given as Needed in Mild Asthma
TREXA: Treating Children to Prevent Exacerbations of Asthma
